# Novel duplication of the cell adhesion molecule L1-like gene in an individual with cognitive impairment, tall stature, and obesity: A case report

**DOI:** 10.3389/fneur.2023.1104649

**Published:** 2023-04-11

**Authors:** Kenny V. Onate-Quiroz, Benjamin Udoka Nwosu, Parissa Salemi

**Affiliations:** Division of Pediatric Endocrinology, Cohen Children’s Medical Center of New York, New Hyde Park, NY, United States

**Keywords:** CHL 1 gene, duplication, autism, tall stature, obesity

## Abstract

The gene that codes for the close homolog of L1 (*CHL1* gene) is located in the 3p26.3 cytogenetic band in the distal portion of the 3p chromosome. This gene is highly expressed in the central nervous system and plays an important role in brain formation and plasticity. Complete or partial *CHL 1* gene-deficient mice have demonstrated neurocognitive deficits. In humans, mutations of the *CHL 1* gene are infrequent with most mutations described in the literature as deletions. This case report describes an individual with a duplication in the *CHL 1* and a presentation consistent with a syndromic form of neurocognitive impairment. To the best of our knowledge, this mutation has not been previously described in the literature.

## Introduction

Mutations in the genes of the distal portion of the short arm of chromosome 3 are rare. The best characterized of these mutations are deletions. Duplications are less frequent and thus, poorly understood. Typically, both deletions and duplications occur *de novo*, although a few familial cases have been characterized, indicating an inheritance pattern (1). The clinical syndrome of these mutations is marked by various degrees of cognitive impairment and dysmorphic features such as trigonocephaly, ptosis, telecanthus, downslanting palpebral fissures, and micrognathia. Implicated genes include *CRBN* (OMIM 609262) and *CNTN 4* (OMIM 607280), which are suggested to cause typical 3p deletion syndrome with associated dysmorphic features. The *CHL 1* gene (OMIM 607416) has been proposed to play an additional role in cognitive impairment, but this is poorly characterized (2–7). In this case report, we describe an individual that contributes to the currently limited literature describing duplications of the *CHL 1* gene and possible association with impaired cognition.

## Case narrative

**FIGURE 1:**
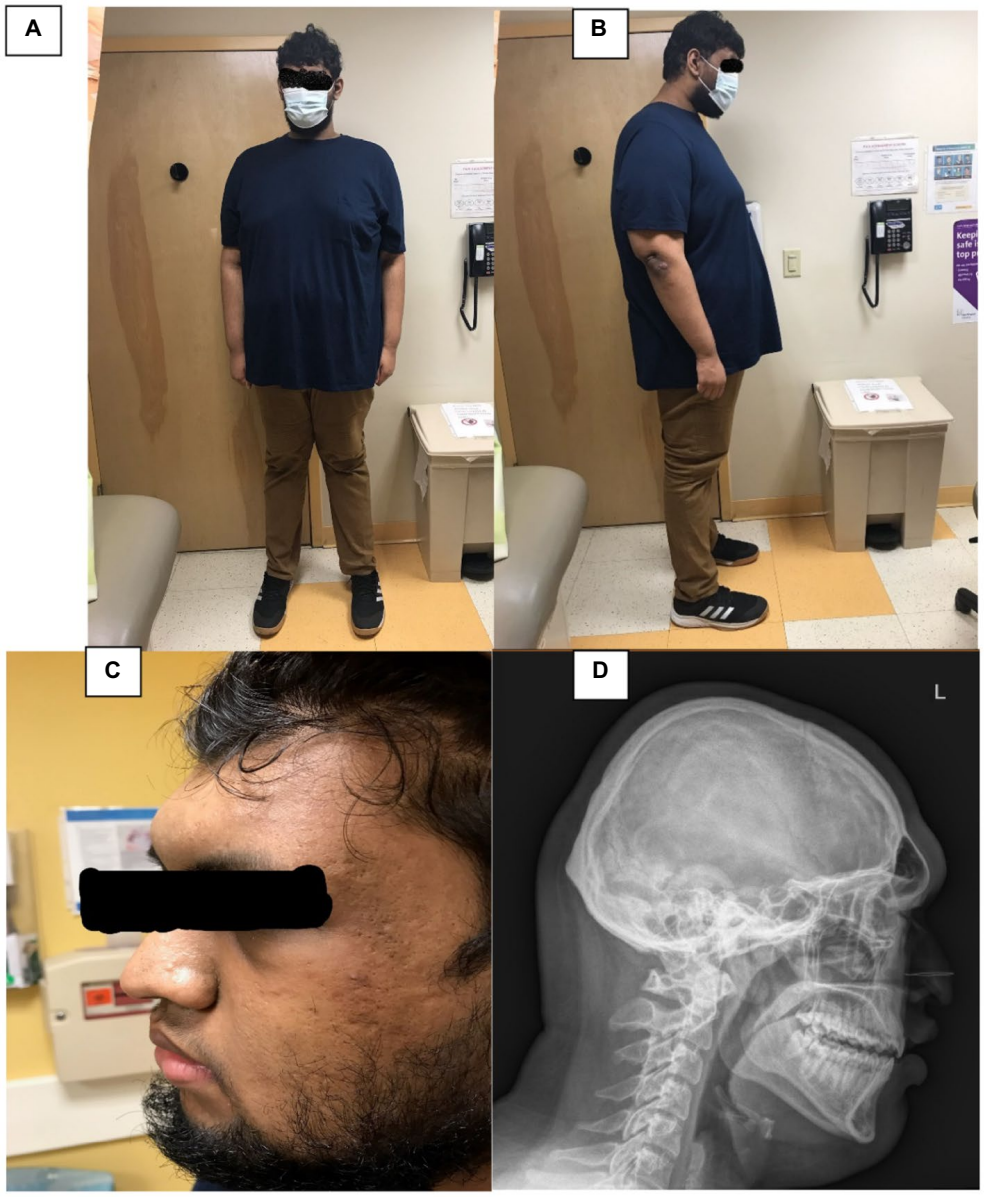
**(A,B)** Depict the patient’s frontal and profile views. **(C)** Depicts a closer view of his face, and **(D)** is a lateraral radiograph of his skull and neck.

**FIGURE 2:**
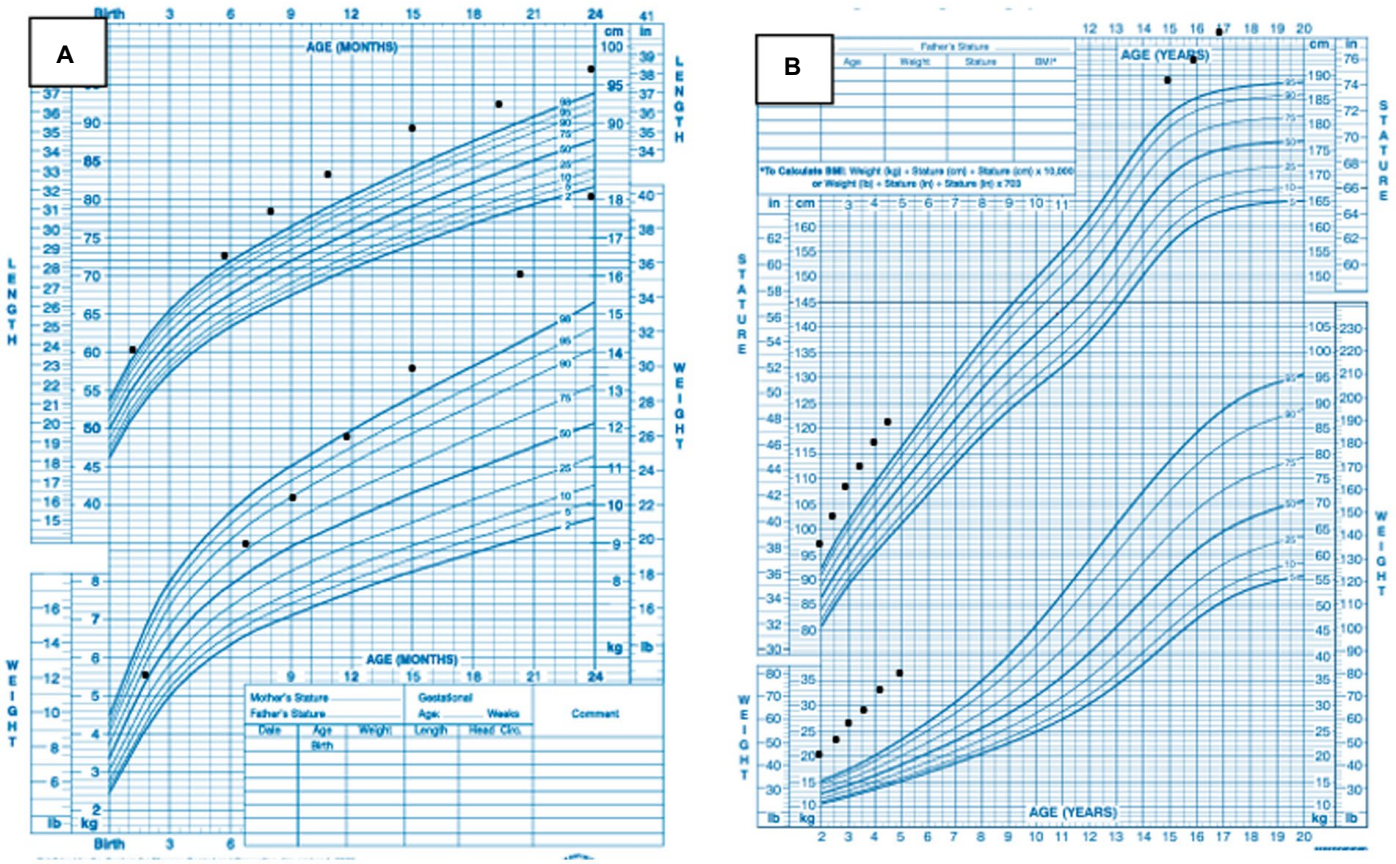
**(A)** Patient’s growth charts from ages 0–24 months. **(B)** Patient’s growth charts from ages 2–17 years.

The patient is a 17-year-old adolescent male of Bangladeshi descent, who was referred to the Pediatric Endocrinology clinic for evaluation for tall stature and abnormal weight gain. He weighed 170.55 Kg and measured at 200.66 cm tall. His BMI was 40.98 Kg/m^2^ (Figures 1A,B, 2B).

He had no significant past medical or surgical history. He was born at term and weighed 3.49 kg and measured 53.34 cm. He achieved normal developmental milestones at the appropriate time, sat at 6 months, walked at 12 months, and spoke his first few words at around 10 months of age. By age 3 years, his parents noted that he was taller than his peers. In kindergarten, he reportedly had difficulty making friends. By the third grade at age 8 years, he was diagnosed with mild autism and received applied behavioral analysis, physical therapy, speech therapy, and occupational therapy as part of his individualized educational plan. A review of his growth chart from his pediatrician’s office showed that his weight reached the 98th percentile at 12 months of age and continued to increase further with age (Figure 2A). His recumbent length reached the 98th percentile at 7 months of age and has remained at >98th percentile (Figure 2A).

He attends regular classes at an age-appropriate grade. He reports difficulty with learning and focusing at school. His grades are mostly in the 60s and 70s. He never repeated a grade but needed to attend summer school following the 9^th^ grade. He continues to receive speech and occupational therapy.

Maternal height is 170 cm, with paternal height at 176.5 cm. He has three brothers and two sisters: a 30-year-old brother who is 177.8 cm and a 77.1 kg, a 28-year-old brother who is 177.8 cm and 90.7 kg, a 26-year-old sister who is 167.6 cm and “average weight,” a 25-year-old sister who is 175.3 cm and 68 kg, and a 20-year-old brother who is 170.2 cm and 68 kg.

Both parents have type 2 DM. There is a male first cousin with autism. There is no family history of overgrowth, abnormal weight gain, intellectual disability, birth defects, or consanguinity. Both parents are from Bangladesh.

At the initial visit, his HgbA1c was 5.7% and thus the reason for referral. He had briefly been in metformin therapy in the past, but it was discontinued. When inquiring about growth and weight gain, father reported that he had grown seven inches in the past 2 years. His review of systems was positive for occasional bilateral knee pain.

On physical examination he was found to be a pleasant, well-appearing, tall, obese young man. There was notable frontal bossing, a prominent occipital bone, and acanthosis nigricans of the neck noted (Figures 1C,D). His hands were notably large for his age. His pubertal exam was consistent testicular volume of approximately 30–35 cc and tanner 5 hair distribution for pubic hair.

Initial laboratory investigations are shown in Table 1. Genetic studies included a karyotype analysis of 46 XY, negative fragile X analysis by PCR and a whole genome chromosomal microarray analysis. Whole genome chromosomal microarray analysis was performed using the Affimetrix CytoScan HD microarray system. The array design is based on hg19. The chromosomal microarray analysis revealed a duplication of 62 Kb of a region within cytogenetic band 3p26.3 (hg 19 genomic coordinates 185,881 – 247,608). The duplicated interval involves the non-coding exon 1 of the *CHL 1* gene and the *CHL 1- AS 2* gene. This was interpreted by the Genetics laboratory as a copy number change of uncertain clinical significance.

**TABLE 1:** Subject’s baseline biochemical parameters.

Parameter	Laboratory value	Normal range
IGF-1	196 ng/mL	131–490
IGFBP 3	4,959 ug/L	2,357–6,319
Growth hormone	0.11 ng/mL	0.03–2.47
Random cortisol	7.5 ug/dL	2.7–10.5
T4	8.0 ug/dL	4.6–12.0
TSH	3.62 uIU/mL	0.5–4.3
Hemoglobin A1c	5.7%	4.0–5.6
Total testosterone	290 ng/dL	264–916
FSH	3.5 mIU/mL	2.6–11.0
LH	4.9 mIU/mL	0.4–7.0

His brain MRI scan showed several small sub-centimeter punctate hypo-enhancing foci on both sides of the pituitary gland, measuring up to 3 mm on the left and 2 mm on the right, concerning for pituitary microadenoma or pituitary cysts. His skeletal survey showed frontal bossing at the level of the frontal sinuses (Figure 1D). The remainder of the survey showed normal osseous structures with fused physes. Neurosurgery evaluation concluded that his brain MRI findings were not related to his gigantism as his hormone profile showed no elevations in either serum growth hormone or insulin-like growth factor 1 concentration. The neurosurgeon recommended a repeat brain MRI scan in 6 months to assess for stability of the lesions.

A 79-gene Prevention Genetics/Rhythm Obesity Gene Panel revealed no pathogenic variants, but two variants of uncertain significance. The first variant is a sequence variant that results in an in-frame deletion and insertion in the *DYRK1B* gene (OMIM 604556). This patient is also heterozygous in the *PLXNA4* gene (OMIM 604280) for a sequence variant that is predicted to result in an amino acid substitution. His genetic evaluation involved molecular studies on his parents for possible mutations. These studies, which were performed by GeneDX for the known duplication in 3p26.3 of uncertain significance, indicated that the 62 Kb duplication of a region within cytogenetic band 3p26.3 was inherited from his mother.

## Discussion

The *CHL 1* gene, located at the chromosomal sub-band 3p26.3 codes for a member of the L1 family of neural cell adhesion molecules (5, 6). The *CHL 1* gene is highly expressed in the central and peripheral nervous system and plays an important role in the structure and functioning of the brain. CHL 1 proteins are involved in axonal migration, synaptic migration, and brain neuroplasticity. CHL 1 regulates neuronal outgrowth, neuronal migration and synapse function (1).

CHL-deficient mice show defects in neurotransmission, behavior, and motor coordination (6, 8, 9). In humans, mutations of the *CHL 1* gene have been associated with distal 3p deletion syndrome (OMIM 613792). This syndrome constitutes a rare contiguous genetic disorder involving the deletion of chromosome 3p25-26 with associated developmental delay (10, 11). Deletions of *CHL 1* gene in have been implicated in a spectrum of neurodevelopmental disorders including autism, intellectual disability, and learning difficulties (12).

A review of the DECIPHER genome database (13) shows that the commonest form of mutation in this *CHL1* gene is deletion, which occurs in 69% of individuals, followed by duplications or triplications that are seen in the remaining 31% of individuals. The commonest pattern on *CHL 1* inheritance is *de novo* in 25% of individuals; 5% are maternally inherited, and 3% are inherited from an unaffected parent as in this case report. Both *CHL 1* deletions and duplications lead to variable degrees of impaired cognitive function (14). Patients with a duplication of the first exon on *CHL 1* gene commonly present with intellectual disability or global developmental delay with or without phenotypic anomalies (14).

The DECIPHER database contains 60 individuals with duplications in the *CHL 1* gene (13). A review of clinical characteristics of these patients showed overlap with those of our patient. Six cases exhibited frontal bossing (n.248616, n.279556, n.392065, n.394069, n.395615, n.401003), One case exhibited obesity (n.399184), one case exhibited increased body weight (n.255856), one case exhibited tall stature (n.255856). Many of these affected subjects exhibited variable forms of cognitive impairment such as intellectual disability, global developmental delay, autism spectrum disorder, learning difficulties, ADHD, and delayed language and speech development. Given the role of CHL 1 in important regulatory functions of the brain, it is reasonable to conclude that *CHL 1* gene duplications will most frequently present with a form of cognitive impairment. Two maternally inherited 189 Kb duplications involving exon 1 of the *CHL 1* gene were found in the database. The first one occurred in an individual with autistic behavior, increased body weight, tall stature, and unknown maternal phenotype (n.355856), while the second one occurred in an individual with delays in fine- and gross motor development, speech and language development, attention deficit hyperactivity disorder, specific learning disability, increased body weight, tall stature, and unknown maternal phenotype (n.355856).

We further reviewed the current literature on CHL 1 beyond what is contained in the DECIPHER database. Earlier descriptions of Individuals with *CHL 1* gene duplications include a girl with intellectual disability and epilepsy who had a maternally inherited duplication (hg 19 genomic coordinates 48,914 – 1,054,209) described by Shoukier et al. (7); and a boy with developmental delay, symptoms of hyperactivity and speech delay who presented a *de novo* duplication (hg 19 genomic coordinates 125,931 – 975,649) as described by Palumbo et al. (12). Li et al. (14) described a male patient with autism spectrum disorder and developmental delay whose duplication (hg 19 genomic coordinates 380,685 – 1,067,787) was transmitted from his unaffected mother, in the same fashion as the patient in this case report. Li et al’s (14) patient’s duplication started at exon 5 and continued through the end of the *CHL 1* gene. Wehii et al. (1) identified a patient with a 3p26.3 microduplication (hg 19 genomic coordinates 190,761 – 349,109) encompassing part of the *CHL 1* gene and the *CNTN6* gene (OMIM 607220) who presented with motor and speech developmental delays and autism (1). These findings are consistent with the hypothesis that *CHL 1* gene duplications result in nonspecific forms of cognitive impairment. The duplicated segment in our patient includes the long non-coding RNA gene *CHL1-AS2* (hg 19 genomic coordinates 237,441 – 239,024). This gene has been suggested as a candidate susceptibility gene in adolescent idiopathic scoliosis (15). It has also been found to be highly expressed in ectopic endometrium tissue in patients with ovarian endometriosis (16). Our literature search did not reveal any previous descriptions of duplications of the *CHL 1-AS 2* gene.

The question regarding the etiology of tall stature and obesity in our patient was examined by performing endocrine biochemical analyses and imaging of the brain. Biochemical markers for growth hormone activity were normal, and while his brain MRI scan revealed an incidental finding of possible sub-centimeter pituitary microadenomas/cysts, it is unlikely that these findings contributed to his tall stature given his normal biochemical analyses. Because our patient’s phenotypical anomalies such as obesity, tall stature, and frontal bossing have been previously described in other individuals with *CHL 1* gene duplications, it is possible that these features are components of a genetic syndrome. Such a syndrome will be conclusively characterized as more individuals with *CHL 1* duplications are described in the literature. Additionally, though these mutations are currently of unknown significance, the *DYRK1B* and *PLXNA4* variants identified in our patient could contribute to his obesity. This is because pathogenic variants in *DYRK1B* gene are associated with autosomal dominant abdominal obesity, while heterozygous missense variants in the *PLXNA4* gene are associated with severe, early-onset obesity (17, 18).

## Conclusion

To our knowledge, this is the first reported case of a duplication of 62 Kb in the 3p26.3 cytogenetic band including the non-coding exon 1 of the *CHL 1* gene in a patient with autism, learning difficulties, and associated phenotypic anomalies such as tall stature, frontal bossing and obesity. Given the limited number of patients with duplications of the *CHL1* gene, it is difficult to conclusively establish that this duplication is the cause of his phenotype. However, his case adds to the limited literature corroborating the hypothesis that duplications of *CHL 1* are associated with syndromic and non-syndromic forms of cognitive impairment. The finding that both deletions and duplications of the *CHL 1* gene result in cognitive impairment suggests that *CHL 1* is a dosage-sensitive gene. More cases are needed to establish genotype–phenotype correlations.

## Data availability statement

The datasets presented in this article are not readily available because of ethical and privacy restrictions. Requests to access the datasets should be directed to the corresponding author.

## Ethics statement

Written informed consent was obtained from the minor (s)’ legal guardian/next of kin for the publication of any potentially identifiable images or data included in this article.

## Author contributions

All authors listed have made a substantial, direct, and intellectual contribution to the work and approved it for publication.

## Conflict of interest

The authors declare that the research was conducted in the absence of any commercial or financial relationships that could be construed as a potential conflict of interest.

## Publisher’s note

All claims expressed in this article are solely those of the authors and do not necessarily represent those of their affiliated organizations, or those of the publisher, the editors and the reviewers. Any product that may be evaluated in this article, or claim that may be made by its manufacturer, is not guaranteed or endorsed by the publisher.
